# Modulation of fear extinction processes using transcranial electrical stimulation

**DOI:** 10.1038/tp.2016.197

**Published:** 2016-10-11

**Authors:** R Abend, I Jalon, G Gurevitch, R Sar-el, T Shechner, D S Pine, T Hendler, Y Bar-Haim

**Affiliations:** 1 School of Psychological Sciences, Tel Aviv University, Tel Aviv, Israel; 2 Functional Brain Center, Wohl Institute for Advanced Imaging, Sourasky Medical Center, Tel Aviv, Israel; 3 Sagol School of Neuroscience, Tel Aviv University, Tel Aviv,Israel; 4 Department of Psychology, University of Haifa, Haifa, Israel; 5 Section on Developmental Affective Neuroscience, National Institute of Mental Health, Bethesda, MD, USA; 6 Faculty of Medicine, Tel Aviv University, Tel Aviv, Israel

## Abstract

Research associates processes of fear conditioning and extinction with treatment of anxiety and stress-related disorders. Manipulation of these processes may therefore be beneficial for such treatment. The current study examines the effects of electrical brain stimulation on fear extinction processes in healthy humans in order to assess its potential relevance for treatment enhancement. Forty-five participants underwent a 3-day fear conditioning and extinction paradigm. Electrical stimulation targeting the medial prefrontal cortex was applied during the extinction-learning phase (Day 2). Participants were randomly assigned to three stimulation conditions: direct-current (DC) stimulation, aimed at enhancing extinction-learning; low-frequency alternating-current (AC) stimulation, aimed at interfering with reconsolidation of the activated fear memory; and sham stimulation. The effect of stimulation on these processes was assessed in the subsequent extinction recall phase (Day 3), using skin conductance response and self-reports. Results indicate that AC stimulation potentiated the expression of fear response, whereas DC stimulation led to overgeneralization of fear response to non-reinforced stimuli. The current study demonstrates the capability of electrical stimulation targeting the medial prefrontal cortex to modulate fear extinction processes. However, the stimulation parameters tested here yielded effects opposite to those anticipated and could be clinically detrimental. These results highlight the potential capacity of stimulation to manipulate processes relevant for treatment of anxiety and stress-related disorders, but also emphasize the need for additional research to identify delivery parameters to enable its translation into clinical practice. Clinical trial identifiers: Modulation of Fear Extinction Processes Using Transcranial Electrical Stimulation; https://clinicaltrials.gov/show/NCT02723188; NCT02723188 NCT02723188.

## Introduction

Research links the processes of fear conditioning and extinction to the treatment of anxiety and stress-related disorders.^1, 2, 3^ Moreover, considerable translational research examines the neural correlates of these processes.^4, 5, 6^ However, research manipulating neural correlates of these processes in humans is still scarce,^7^ which limits basic clinical integration. To address the need for such work, the current study examines the effects of electrical brain stimulation on processes associated with fear extinction in healthy humans.

Exposure therapy, involving gradual extinction of maladaptive fear associations, represents one of the most effective treatments for anxiety and stress-related disorders.^8, 9, 10^ In this treatment approach, stimuli associated with a fear response are evoked in a safe context, leading to the formation of new safety associations.^11^ However, many patients either fail to respond or exhibit spontaneous symptom relapse following treatment,^9, 12^ creating a need to develop methods for enhancing extinction learning to improve therapeutic effects.^3, 11, 13^

Transcranial electrical stimulation (tES) is an emerging technique that transiently influences brain activity.^14, 15, 16^ tES delivers current primarily in two modes. Direct current (DC) applies steady current (typically 1–2 mA), which may yield memory-enhancing effects associated with long-term potentiation (14). In contrast, alternating current (AC) is applied at a specific frequency, which affects cortical oscillations.^16, 17^

To date, tES research has focused predominantly on modulating cognitive or motor functions.^17, 18, 19, 20, 21, 22, 23^ However, to the best of our knowledge, tES has not been applied to enhance learning processes associated with fear extinction.^13^ On the basis of brain imaging and basic science research, one promising tES approach might attempt to facilitate extinction learning and consolidation, resulting in the formation of more robust safety associations.^13, 24, 25^ Support for this approach derives from neuroimaging studies on ventromedial prefrontal cortex (vmPFC) in extinction, posttraumatic stress disorder, and anxiety disorders.^4, 11, 26, 27, 28, 29, 30^ Preliminary support for this proposition comes from a study demonstrating that stimulation of rodent vmPFC analog enhanced fear extinction.^31^

An alternative approach might attempt to disrupt the reconsolidation of the fear memory trace following its reactivation during extinction.^32, 33, 34, 35, 36^ Reactivation of a fear memory trace is associated with increased neural coupling between dorsal mPFC regions and the amygdala.^37, 38, 39, 40, 41^ As low-frequency oscillations within cortical networks may reflect processes similar to long-term depression,^42, 43, 44, 45^ low-frequency AC stimulation of the dorsal mPFC could produce an analogous effect, thereby disrupting reconsolidation of an activated fear memory during extinction.

The present study tests the effects of these two approaches using a fear conditioning and extinction paradigm conducted over three consecutive days. Skin conductance response (SCR) and self-reports assessed fear. tES was administered during the extinction phase (Day 2) via an electrode alignment targeting the medial prefrontal cortex, and we compared the effects of the two active stimulation conditions on extinction-related processes in terms of successful subsequent reduction in spontaneous fear response recovery (Day 3). Thus, the first group received DC stimulation during the extinction phase aimed to increase vmPFC activation, and thereby facilitate extinction learning. The second group received AC stimulation, aimed to disrupt reconsolidation of the activated fear memory trace by inducing a low-frequency current during extinction. A third group received sham stimulation. We hypothesized that relative to the sham group, the active stimulation modes would modulate processes associated with fear extinction and fear response recovery.

## Materials and methods

### Participants

Forty-five healthy participants (21 females; mean age=25.2 years, s.d.=5.7) were recruited and randomly assigned to three experimental conditions (see below). Groups did not differ in age or gender, *P*-values>0.11. Participants received $75 for their effort. All participants provided written informed consent. The study protocol was approved by Tel Aviv University and Tel Aviv Sourasky Medical Center Ethics Committees, and conformed to the Code of Ethics of the World Medical Association (Helsinki Declaration). All participants completed a screening questionnaire to ascertain that they did not report any neurological or psychiatric disorders, or other contraindications to tES (for example, metal or cochlear implants, recent consumption of alcohol, coffee or other psychotropic drugs or medicine).^14^ Sample size was based on previous research on laboratory manipulation of similar processes in healthy participants.^32, 46^ One participant from the Sham stimulation condition discontinued participation after one session, and her data were excluded from analyses.

### Fear conditioning and extinction task

**Figure 1:**
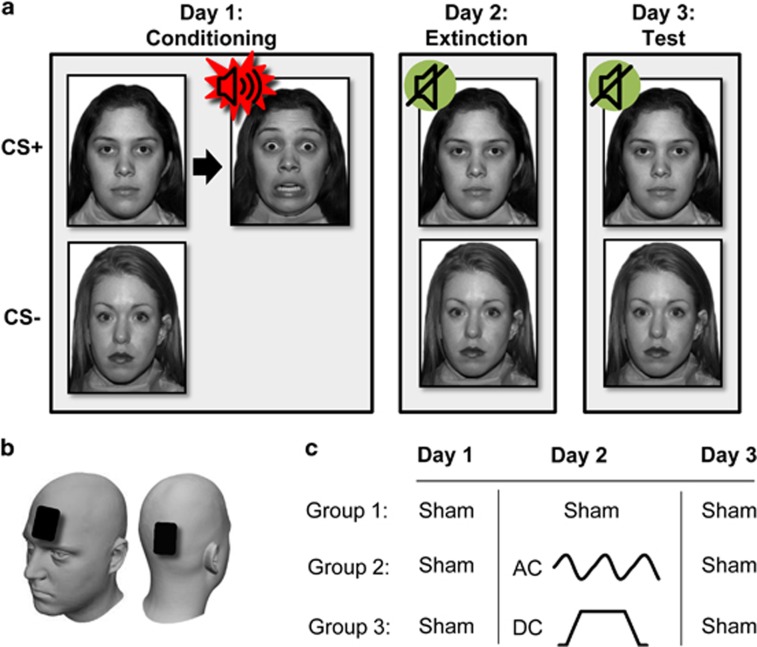
(**a**) A schematic depiction of the conditioning, extinction and test phases of the paradigm. Face images are from the NimStim set.^46^ (**b**) Alignment of stimulation electrodes; the frontal electrode was the anode. (**c**) The three stimulation conditions in the study differed in terms of stimulation during Day 2 (extinctions): sham, AC and DC. AC, alternate current; CS, conditioned stimulus; DC, direct current.

Participants completed a 3-day fear conditioning and extinction task (Figure 1a) adapted from previous research.^4, 47^

#### Stimuli

Photographs of a light-haired woman and a dark-haired woman displaying neutral, closed-mouthed neutral expressions (NimStim set: 01 F, 03 F)^48^ served as the differential conditioned stimuli (CS+ and CS−, counterbalanced across participants). The CS− was presented for 8 s. The CS+ was presented for 7 s, and predicted the unconditioned stimulus, which consisted of the same actor displaying an open-mouthed fearful expression paired with a loud female scream (presented over computer speakers at 80 dB SPL) for 1 s.

#### Task description

Day 1 started with a pre-conditioning phase during which each CS was presented four times with an intertrial interval randomly ranging between 10 and 20 s. Next, during the conditioning phase, each CS was presented 10 times. On 80% of CS+ trials, the CS+ (7 s) was paired with the unconditioned stimulus (1 s). The CS− (8 s) was never followed by a unconditioned stimulus. Participants were instructed that they could learn to predict when the UCS would occur, but were not informed of the CS/UCS contingency. During the extinction phase (Day 2), the CS+ and CS− were presented (10 trials of each CS) in the absence of the unconditioned stimulus, except for the first CS+ trial, which was reinforced and served as a reminder of the conditioned association from Day 1. Of note, the conditioning and extinction phases took part in different days,^32, 46^ to allow the newly acquired fear memory to undergo consolidation before extinction, thus allowing the paradigm to better serve as a model for exposure therapy, which typically is given a while after trauma originally occurred.^5^ In addition, given that some of the potential tES effects target reconsolidation, it was imperative to ensure that such processes occur following fear acquisition. Day 3 served as the test phase, and consisted of eight non-reinforced presentations of each CS. In all task phases, the order of CS presentations was pseudorandomized (two different orders counterbalanced across participants and groups). The task was programmed using the E-Prime 2.0 software (Psychology Software Tools, Pittsburgh, PA, USA).

### Skin conductance response

SCR was measured continuously using two conductive-gel-coated electrodes placed on the medial phalanx of the ring and middle fingers of the non-dominant hand.^49^ Electrodes were connected to an amplifier (BrainAmp ExG MR and GSR Sensor non MR, Brain Products, Gilching, Germany), and recorded via the Brain Vision Recorder software (Brain Products), at a sampling rate of 250 Hz.

### Electrical stimulation

tES was applied using a battery-powered stimulator (DC-Stimulator Plus, NeuroConn, Ilmenau, Germany). Two 5 × 7 cm rubber electrodes coated with conductive gel were used, whereby one acted as the anode electrode, increasing neuronal excitability and activity, and the other as the cathode return electrode, decreasing excitability and activity.^14, 16^ An electrode alignment targeting the medial prefrontal cortex was used (Figure 1b). The anodal electrode was placed centrally over the forehead, with its lower edge at the nasion line, in line with previous studies targeting the mPFC,^50, 51^ whereas the reference electrode was placed under the occipital bone. Current was ramped up (30 s) and ramped down (30 s) before and after stimulation, respectively.

Participants were randomly assigned (using an in-house randomization software) into one of three stimulation conditions: (1) Sham stimulation (current ramped up and then immediately ramped down, and then maintained at 0 mA); (2) DC (constant current: 1500 mA, duration: 20 min); or (3) AC (sinusoidal waveform with frequency: 1 Hz, duration: 20 min). During the conditioning (Day 1) and test (Day 3) phases, all participants received sham stimulation. To examine the effect of electrical stimulation on extinction processes, on Day 2 participants received stimulation according to their assigned condition (Sham, DC or AC). Participants and experimenters were blind to the stimulation condition allocation. The same alignment was used in the two active stimulation conditions as in both cases mPFC regions were targeted, whereas the different stimulation modes targeted different neural mechanisms.

### Questionnaires

#### Self-reported fear

At the end of each session, participants were shown (on paper) the CS+ and CS− and asked to indicate how afraid they were when they saw each of the faces. Ratings were on a 10-point Likert scale (1=not afraid, 10=extremely afraid), and were collected after each phase.

#### Stimulation debriefing

A stimulation-debriefing questionnaire was administered at the end of each session to assess potential confounding effects related to sensing the stimulation. Participants were asked to indicate: (1) how uncomfortable they felt during the session (0=none to 10=extremely uncomfortable); (2) whether they sensed any stimulation during the session (yes/no); and (3) whether they felt any side effects during the session that they believed were associated with stimulation.

#### Psychological inventories

Three self-reported questionnaires were administered before the start of the first session: trait anxiety (Spielberger State-Trait Anxiety Inventory),^52^ depression (Beck Depression Inventory)^53^ and past experienced trauma.^54^ The three groups did not differ on these instruments, *P*-values>0.14, suggesting that the effects observed in this study could not be alternatively explained by differences in these factors.

### Procedure

All task sessions were conducted in three consecutive days using the same experimental context (time of day, room, and light, sound and temperature conditions). On Day 1, before the fear conditioning task, participants were briefed on the methods used in the experiment, including safety aspects and potential discomfort. Participants then provided signed informed consent and completed the psychological questionnaires. Each session started by applying the skin conductance and tES electrodes. Following each session, participants completed the stimulation-debriefing and explicit fear assessment questionnaires.

### Data analysis

#### Skin conductance response

Raw SCR data were first downsampled to 1  Hz using EEGLAB.^55^ Continuous decomposition analysis was then performed using Ledalab, separating raw data into phasic and tonic components.^56^ Analysis was then performed on the phasic component of the data.^57^ For each experimental event (CS+, CS−) the trough-to-peak value (maximal SCR value minus minimal SCR value) within the 1–7-s post-stimulus onset time window was calculated and then square-root-transformed.^4, 58^

Fear conditioning was assessed using a repeated-measures analysis of variance (ANOVA) on SCR levels with CS (CS+, CS−) and Trial (trials 1–10 in the conditioning phase, Day 1) as within-subject factors. The effect of stimulation on extinction was assessed using a repeated-measures ANOVA on SCR levels with CS (CS+, CS−) and Trial (extinction phase trials 2–10, Day 2) as within-subject factors, and Stimulation (Sham, DC and AC) as a between-subject factor. The effect of stimulation on retention of acquired fear following extinction was assessed using a repeated-measures ANOVA on SCR levels in the first trial of the test session (Day 3; 32), with CS (CS+,CS−) as a within-subject factor and Stimulation (Sham, DC and AC) as a between-subject factor. No significant differences in baseline tonic SCR levels were observed between the three groups before the conditioning and the extinction phases, *P*-values>0.65, indicating no *a priori* group differences.

#### Self-reported fear

Assessment of self-reported fear of the CS+ and CS− following each task phase was conducted using separate repeated-measures ANOVA on fear ratings. Thus, following the conditioning phase, CS (CS+, CS−) served as a within-subject factor, whereas Stimulation (Sham, DC and AC) was added as a between-subject factor in analyses following the extinction and extinction test phases.

Significant effects were detected at *α*=0.05 level. All tests were two-sided. The mean CS levels and self-reported fear levels per day and group did not significantly differ from the normal distribution, *P*-values>0.05 (corrected for multiple tests), permitting the use of parametric tests; in addition, variances of these variables did not significantly differ between the groups, *P*-values>0.05 (corrected for multiple tests).

## Results

### Skin conductance

**Figure 2:**
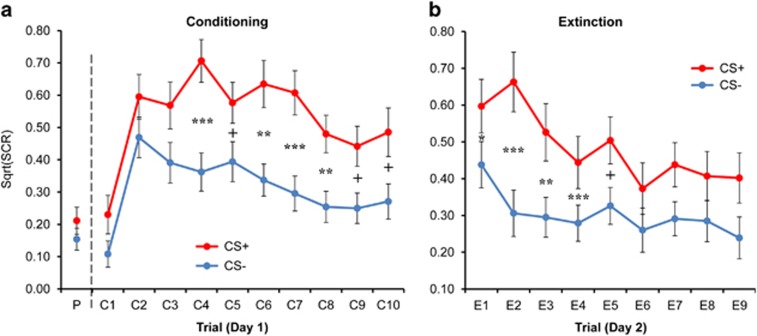
The mean skin conductance response (SCR) for each CS+ and CS− in each trial of the (**a**) conditioning phase (C1–C10, Day 1) and (**b**) extinction phase (E1–E9, Day 2). CS, conditioned stimulus; P, pre-conditioning phase. Asterisks represent significance of paired-samples *t*-tests (comparing CS+ and CS− in each trial); ^+^*P*<0.1; **P<0.01; ***P<0.001. Error bars indicate s.e.m.

During pre-conditioning (Day 1), no significant difference was detected in SCR between CS+ and CS−, *t*(43)=0.55, *P*=0.59. During the subsequent conditioning phase (trials C1–C10, Figure 2a), a repeated-measures ANOVA revealed a significant main effect of CS on SCR levels, F(1,43)=46.1, *P*<0.001, with a greater response to the CS+ than to the CS−, thereby confirming differential fear conditioning. Paired-samples *t*-tests further showed that the response to the CS+ was greater than to the CS− from the fourth acquisition trial and onward, all *P*-values ranging from <0.001 to 0.098 (Bonferroni-corrected for repeated comparisons). In addition, we observed a main effect of Trial, F(9,387)=10.0, *P*<0.001.

During the extinction phase (Day 2, Figure 2b), a repeated-measures ANOVA yielded a significant CS × Trial interaction, F(8,328)=2.26, *P*=0.023, suggesting differential responses to the different CS types through the extinction phase. Follow-up paired-samples *t*-tests showed that the conditioning effect (CS+>CS−) was maintained for the first half of the extinction phase, but diminished with time, and became nonsignificant (*P*-values>0.05) from the fifth trial and onward, indicating extinction of the learned fear response. Importantly, no stimulation effects were observed, *P*-values>0.37, suggesting that stimulation did not immediately affect responding.

**Figure 3:**
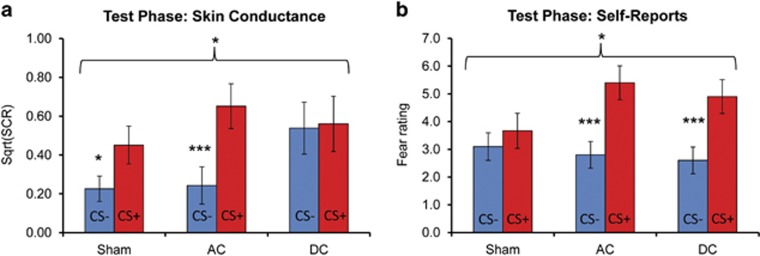
Extinction test phase (Day 3). The mean (**a**) skin conductance response and (**b**) explicit fear ratings for CS+ and CS− in each stimulation condition (Sham, AC and DC). Note: *P<0.05; ***P<0.001. AC, alternating current; CS, conditioned stimulus; DC, direct current; SCR, skin conductance response. Error bars indicate s.e.m.

Retention of SCR-based fear response following extinction was assessed using SCR levels in the first trial on Day 3 (extinction recall; for a description of SCR levels across the session, see Supplementary Figure S1 in the Supplementary Online Material). A repeated-measures ANOVA on SCR levels revealed a significant Stimulation Group × CS interaction effect, F(2,41)=3.83, *P*=0.030 (Figure 3a). Follow-up analyses revealed retention of fear in the Sham condition, *t*(13)=2.36, *P*=0.034, and in the AC condition, *t*(14)=5.09, *P*<0.001. However, in the DC condition the response to the CS− was in a comparable magnitude to the response to the CS+, *t*(14)=0.19, *P*=0.852.

### Self-reported fear

Following the conditioning phase, we observed a significantly greater mean fear rating to the CS+ relative to CS−, F(1,43)=87.66, *P*<0.001. Following extinction, a main effect of CS was still observed, F(1,40)=33.50, *P*<0.001, but no effects of Stimulation were noted. Fear ratings to the CS+ following extinction were reduced relative to their levels following conditioning, *t*(43)=4.44, *P*<0.001 (paired-samples *t*-test).

At the test phase, a significant Stimulation Group × CS interaction was observed, F(2,41)=3.87, *P*=0.029 (Figure 3b). Follow-up analyses revealed retention of fear in the AC condition, *t*(14)=4.33, *P*<0.001, and in the DC condition, *t*(14)=4.49, *P*<0.001, but not in the Sham condition, *t*(13)=1.10, *P*=0.29.

### Stimulation debriefing

No significant differences between the stimulation conditions were observed in terms of participants' reports of sensing the stimulation, *χ*^2^=0.47, *P*=0.79, or discomfort ratings, F(1,41)=0.08, *P*=0.92, when stimulation was applied.

## Discussion

The results of the current study demonstrate that tES applied during extinction learning modulated fear response during a subsequent test phase. However, unexpectedly, AC stimulation potentiated the expression of fear response following extinction, whereas DC stimulation led to overgeneralization of the fear response to non-reinforced stimuli.

We hypothesized that DC stimulation intended to target that the mPFC would enhance fear extinction learning; our results suggest that this stimulation condition led to potentiation and generalization of fear during the test phase. The surprising direction of our findings could reflect a complex role for vmPFC function during extinction, such that inducing enhanced activation continuously during extinction learning may not directly translate into enhanced extinction retrieval. Other work also suggests such complexity. For example, Milad *et al.*^31^ demonstrated in rodents that the specific timing of electrical stimulation administration, relative to CS presentation during extinction (for example, 1 s pre-CS or 0.1 s post-CS), is critical for its later behavioral expression, suggesting that time-selective vmPFC activation may underlie effective extinction. Thus, future research may consider using protocols featuring time-locked stimulation bursts in order to influence such time-selective processes. Alternatively, stimulation targeting mPFC may have also modulated activity in additional brain regions associated with fear processing. For example, increased activation in dorsomedial regions implicated in the mediation of fear responses,^37, 38, 39, 59^ concurrently with the presentation of both CS+ and CS− during extinction, may have led to enhanced association of both these stimuli with fear response circuitry, leading to diminished fear extinction and overgeneralization of fear.^60, 61^

We also hypothesized that low-frequency AC stimulation would interfere with reconsolidation of the fear memory trace. The observed potentiation of fear response, in terms of retention of skin conductance and self-report fear responses, following AC stimulation, suggests an opposite effect. Whereas previous research on non-human primates finds low-frequency stimulation to disrupt reconsolidation of fear memories,^42^ this work relies on deep-brain stimulation of analog brain regions in animals. Such invasive methods may produce different effects than the methods used in the current study, generating a less specific topographical effect,^62, 63^ resulting in the observed unexpected effects.

With regard to the potential utility of tES for the enhancement of exposure therapy, our results indicate that extinction of learned fear is indeed susceptible to modulation via electrical stimulation targeting the mPFC. These novel findings extend a previous demonstration of the effect of offline administration of transcranial magnetic stimulation on extinction processes.^7^ However, under the stimulation parameters tested here (DC and low-frequency AC stimulation targeting the mPFC), learning was modulated in ways that are opposite to those anticipated and could be detrimental from a clinical standpoint. Future research should strive to identify an optimal set of stimulation parameters in terms of enhancing fear extinction processes. In a broader sense, despite its relatively recent emergence, tES has been rapidly applied in the treatment of psychiatric conditions, aiming to reduce symptomatology by rectifying maladaptive patterns of brain activity. Promising results have been reported for major depression, Alzheimer's disease, Parkinson's disease, schizophrenia, stroke and addiction.^13, 19, 21, 64, 65, 66^ However, our results indicate that the effects of tES on brain activity may potentially yield adverse results in the context of treatment outcome. That is, tES may potentially not only be non-beneficial for patients in the context of fear extinction-based treatments, but in some cases might exacerbate and overgeneralize their anxiety condition. Thus, the application of tES in a clinical context should be done with caution, and only following rigorous preclinical testing.

In addition, whereas the effects produced under the current stimulation parameters could be detrimental in terms of treatment application, they do model a malignant state that by itself could be useful for further treatment development. For example, overgeneralization of fear is a defining feature of anxiety disorders.^60, 61^ The current results suggest that DC stimulation can generate this effect in healthy participants, mimicking the pathological symptom and thus providing a model on which the efficacy of novel interventions could be tested.

Finally, it may be noted that in some of the stimulation conditions, the different measures of fear retention (SCR and self-reports) did not correspond. Fear acquisition and extinction in human subjects are frequently indexed using implicit physiological arousal measures (such as SCR) and explicit verbal self-report measures; inconsistencies between these measure types are common.^67, 68^ Such differences may arise from specific aspects of fear expression that each type gauges, such that the former may reflect subtle, automatic physiological changes in response to a stimulus, whereas the latter involves the conscious interpretation of such arousal. The observed group differences in explicit and implicit fear response patterns suggest that different stimulation modes may yield distinct effects on autonomic and subjective measures of learned fear.

Naturally, the current study does not come without limitations. First, the application of tES without the use of imaging methods limits the ability to directly attribute observed behavioral and physiological effects to a specific neural function targeted by stimulation.^69^ Complementing brain stimulation methods with neuroimaging methods (for example, functional magnetic resonance imaging or electroencephalography) can usefully guide research in this field by allowing researchers to better target specific neural regions and mechanisms by empirically testing different stimulation parameters. Such methodology may aid in causally inferring about mechanisms and dynamics of brain activity underlying specific cognitive processes or symptomatology, and in designing targeted therapeutic interventions.^69^ Second, as noted, tES application may yield a distributed effect on brain activity, potentially leading to unintended effects. The use of smaller, 'high-definition' electrodes and current-flow modeling in future research may increase the spatial focality of the delivered current,^70, 71^ and thus potentially target specific regions with greater accuracy. Likewise, future studies may also wish to use an extracephalic location of the return electrode in order to rule out its potential contribution to observed effects. Finally, the current study was conducted on a non-selected population. Whereas conducting initial assessment of treatment feasibility on such a population is a logical first step in treatment development, it is possible that the outcomes of the study would be different in a clinical population, in light of the aberrant nature of the targeted neural processes.^1, 2, 3^

In conclusion, the current study demonstrates the capability of tES to modulate fear extinction processes. However, additional research is still needed in order to identify delivery parameters that would enable the translation of this capability into clinical practice.
