# Supplementary material for: Modulation of fear extinction processes using transcranial electrical stimulation
Source: Transl Psychiatry. 2016 Oct 11;6(10):e913–. doi: 10.1038/tp.2016.197 (PMC5315554; doi:10.1038/tp.2016.197)
Supplement: Supplementary Figure legend [file tp2016197x1.doc]

**Modulation of fear extinction processes using transcranial electrical stimulation**

Rany Abend, Itamar Jalon, Guy Gurevitch, Roy Sar-el, Tomer Shechner, Daniel S. Pine, Talma Hendler, & Yair Bar-Haim

**Supplementary online material – Figure S1 caption**

**Figure S1.** Mean skin conductance response (SCR) for the CS+ and CS- in each trial of the test phase (T1-T8, Day 3), per stimulation condition (Sham, AC, DC; applied in Day 2).

*Note*: CS = conditioned stimulus, AC = alternating current, DC = direct current. Error bars indicate SEM.
