# Supplementary figures and images for: Modulation of fear extinction processes using transcranial electrical stimulation
Source: Transl Psychiatry. 2016 Oct 11;6(10):e913–. doi: 10.1038/tp.2016.197 (PMC5315554; doi:10.1038/tp.2016.197)

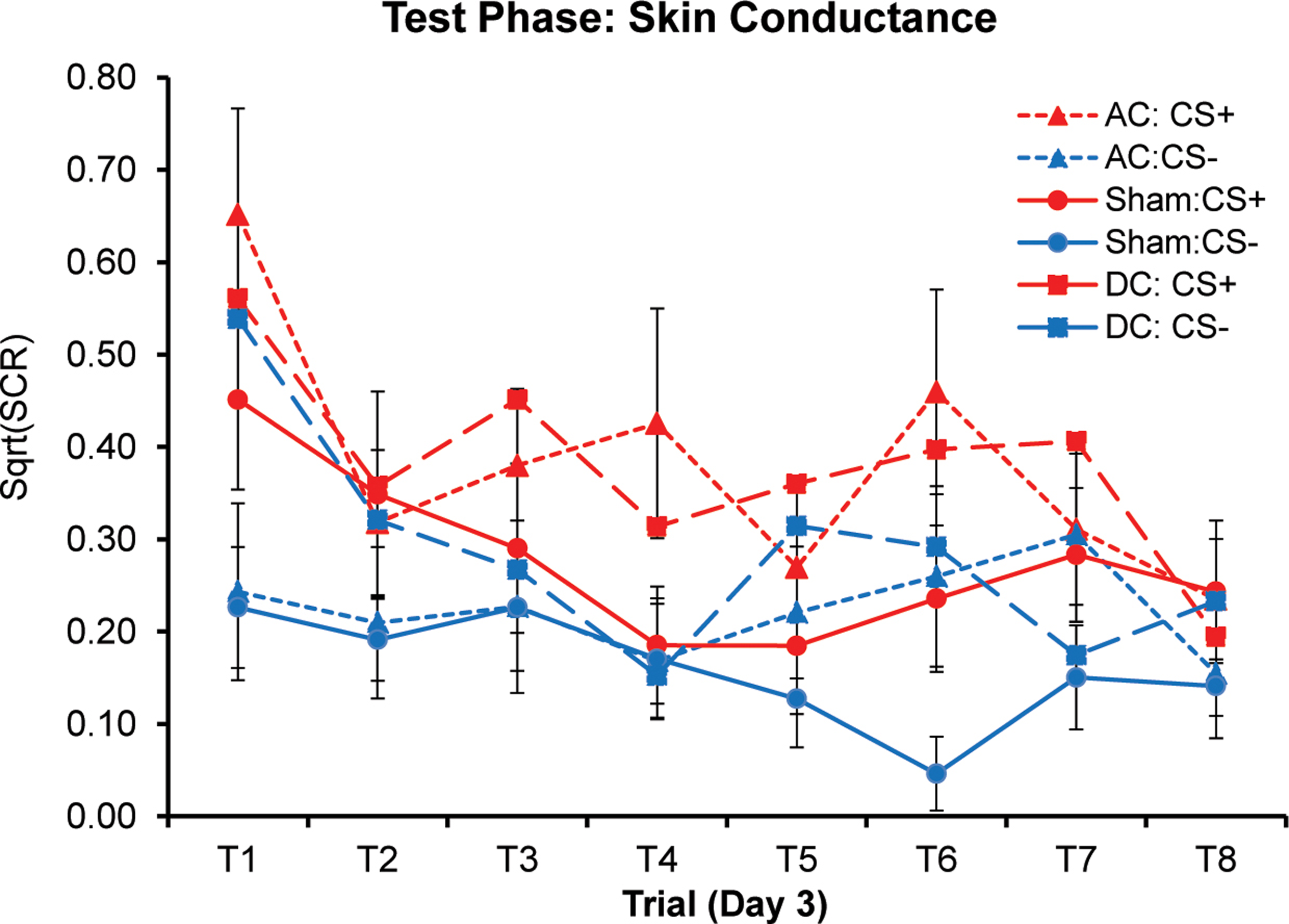

Supplement: Supplementary Figure 1 [file tp2016197x2.tif]
